# Wafer Type Ion Energy Monitoring Sensor for Plasma Diagnosis

**DOI:** 10.3390/s23052410

**Published:** 2023-02-22

**Authors:** Chansu Han, Yoonsung Koo, Jaehwan Kim, Kwangwook Choi, Sangjeen Hong

**Affiliations:** 1 Department of Electronics Engineering, Myongji University, 116 Myongjiro, Yongin 17058, Gyeonggi, Republic of Korea; 2 Future Business Division, Fine Semitech Corperation, Hwaseong 18487, Gyeonggi, Republic of Korea

**Keywords:** wafer-type sensor, monitoring sensor, ion energy, plasma diagnosis, in situ monitoring, ion current

## Abstract

We propose a wafer-type ion energy monitoring sensor (IEMS) that can measure the spatially resolved distribution of ion energy over the 150 mm plasma chamber for the in situ monitoring of the semiconductor fabrication process. The IEMS can directly be applied to the semiconductor chip production equipment without further modification of the automated wafer handling system. Thus, it can be adopted as an in situ data acquisition platform for plasma characterization inside the process chamber. To achieve ion energy measurement on the wafer-type sensor, the injected ion flux energy from the plasma sheath was converted into the induced currents on each electrode over the wafer-type sensor, and the generated currents from the ion injection were compared along the position of electrodes. The IEMS operates without problems in the plasma environment and has the same trends as the result predicted through the equation.

## 1. Introduction

The plasma process has become widely used in manufacturing semiconductors, displays, and electronic devices as semiconductor manufacturing technology has advanced. Plasma has an anisotropic property that helps it form narrow and deep patterns [1]. As a result, interest in dry etching capable of anisotropic etching for high integration and miniaturization has grown, and plasma has emerged as a key component of semiconductor processing technology. On the other hand, specifications for plasma damage, etch selectivity, critical dimension control, and etch uniformity have become more demanding and challenging.

Plasma etching is essential in semiconductor fabrication to reduce device size and increase the aspect ratio of etched features. Plasma uniformity has become the most critical variable in ensuring the reproducibility and stability of the process and increasing yield as plasma has become the leading technology of the semiconductor manufacturing process. The ion current in the plasma parameters affects etching uniformity, and the ion energy on the substrate surface determines etching selectivity and rate [2,3,4,5]. Plasma damage caused by high-energy ion bombardment, on the other hand, may cause substrate damage. Such damage reduces the device’s reliability and lifetime [6,7,8]. As a result, precise control of ion energy is required to achieve optimal process results.

The importance of technology for diagnosing process state grows in direct proportion to the difficulty of the process and the degree of integration required. Several studies have been conducted to assess the current state of the semiconductor process. The most common process diagnostic techniques are optical, electrical, and chemical methods. One of the optical diagnostic methods, optical emission spectroscopy, can monitor the plasma state by separating the plasma state into wavelengths [9,10,11]. It is primarily used in the etching process for end-point detection. Furthermore, the optical plasma monitoring system (OPMS) [12,13] is a diagnostic method for monitoring the total amount of plasma light at high speed. This optical process diagnostic method, on the other hand, optically measures the total distribution of plasma. A chemical diagnostic method, the quadrupole mass spectrometer, evaluates the state of a process by analyzing the amount of residual gas generated after the process [14,15,16]. The VI Probe measures impedance within the chamber by connecting the sensor to the semiconductor radio frequency (RF) generator, matcher, and chamber [17,18,19]. Because the above four types of sensors have limitations, measuring the process distribution in the wafer state in an actual plasma process is difficult.

By inserting the LP’s probe directly into the chamber, plasma parameters can be measured position by position [20,21,22]. The disadvantage, however, is that the process gas is limited to Ar and the probe must be directly inserted into the chamber, which acts as a limitation that is difficult to apply to the actual process [23,24]. Furthermore, because the actual process is performed on a substrate, the development of process monitoring technology on the substrate is required.

A wafer-type sensor, called on-wafer sensor, has been researched and developed for this purpose to measure process uniformity on the wafer surface [25]. A sensor that can diagnose and measure process parameters in the same shape as a real wafer is known as an on-wafer sensor. By inserting the sensor inside the wafer or manufacturing the sensor in the form of a wafer, this enables process diagnosis and data acquisition under the same or similar conditions as the actual process wafer. KLA’s SensArray^®^ and Impedans’ Semion RFEA are two current on-wafer sensor vendors (Retarding Field Energy Analyzer). As the importance and demand for semiconductor process diagnosis sensors grow, research is being conducted on an on-wafer platform that combines two or more types of sensor technologies, rather than just one.

We fabricated a wafer-type ion energy-monitoring sensor (IEMS) in this study. The wafer-type IEMS is a sensor that monitors ion energy and is designed in the form of a wafer. By inserting it into semiconductor manufacturing equipment, it is possible to monitor the ion energy generated in the same environment as the actual semiconductor manufacturing process. Furthermore, unlike previous probe-type measurement equipment, it can be inserted into a plasma bulk to improve plasma interference and diagnose a substrate on which an actual process occurs.

## 2. IEMS

### 2.1. Principle of IEMS and Ion Energy

**Figure 1:**
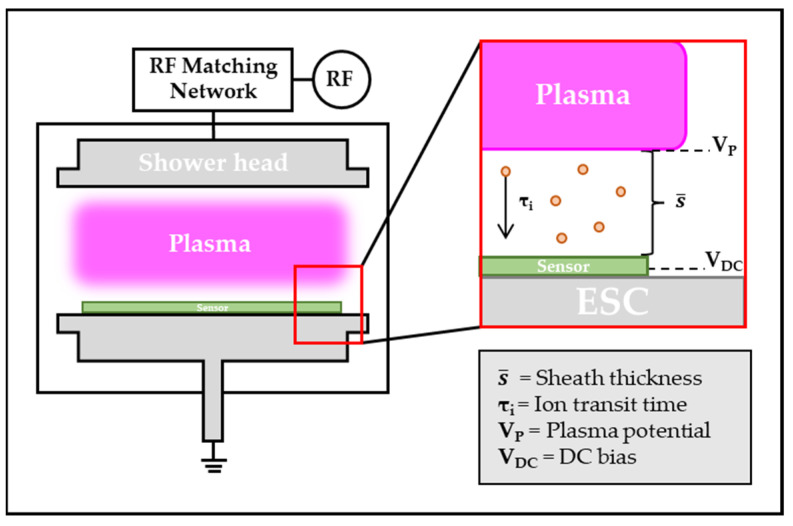
Components of plasma for calculating ion energy.

The wafer-type IEMS is a sensor that detects plasma states by converting ion current generated during the plasma process into ion energy. The current flowing on the substrate is detected by the magneto-impedance (MI) sensor when current is generated in the plasma process with the data compared using the LP, a sensor that can calculate the ion current, to calculate the ion current from the current measured in the IEMS (Figure 1).

Ion energy may be calculated by measuring electron density and ion density in the bulk plasma through the Langmuir probe (LP). Measure the current by placing the sensing pad and LP’s tip of IEMS on the same x-axis. Through this process, a constant for converting the current generated in the plasma into an ion current is obtained.

The ion current measured by the IEMS is as follows:

(1) $${\bar{J}}_{i}=0.6en_{e}\sqrt{\frac{kT_{e}}{m_{i}}}\to \alpha I_{C}\;$$

In collisionless RF plasma sheaths, the shape of the IED is determined by the ratio of ion transfer time to the RF cycle. The ion transfer time is the time it takes for the ion to cross the sheath, which can be calculated using the Child–Langmuir law. First, to calculate the ion transfer time, the thickness of the coating is calculated as follows [26]:

(2) $$\bar{s}=\frac{2}{3}{\left(\frac{2e}{M_{i}}\right)}^{\frac{1}{4}}\;{\left(\frac{\varepsilon_{0}}{{\bar{J}}_{i}}\right)}^{\frac{1}{2}}{\bar{V}}_{s}{}^{\frac{3}{4}}\;$$

where e is the electronic charge, $M_{i}$ is the ion mass, $\varepsilon_{0}$ is the vacuum permittivity, and ${\bar{J}}_{i}$ is the ion current density in the sheath. The pressure range, where the etch process is mainly performed is the collisionless section where the ion distribution is determined by the $\tau_{i}$*/*$\tau_{rf}$. The ion transit time is then obtained using the following equation [6,27]:

(3) $$\tau_{i}=3\bar{s}\sqrt{\frac{M_{i}}{2e\hat{V}s}}\;$$

Combining the above formulas, the ion energy can be calculated as follows [28]:

(4) $$∆E=\frac{2eV_{pp}}{\pi }\left(\frac{\tau_{rf}}{\tau_{i}}\right)\;$$

### 2.2. The Design of IEMS

To minimize plasma interference, a wafer-type IEMS was designed as a printed circuit board (PCB) of the same size as a 6-inch wafer and consists of four layers. This improves the issue of being inserted directly into plasma and does not damage the measuring component. Since the IEMS is located in the chuck where the plasma process occurs, it does not directly affect plasma properties and conditions. Furthermore, if it meets the same thickness and impedance requirements as silicon wafers, it is possible to collect data in the same environment as the actual process.

**Figure 2:**
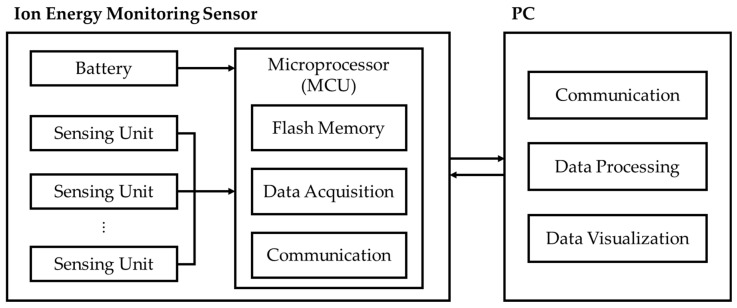
Block diagram of IEMS.

IEMS includes a sensing section, a control section, and a charging section. When data measurement is initiated, the current signal is detected by the MI sensor in the sensing section. A built-in amplifier and analog-to-digital converter convert the current signal detected by the sensing unit into a digital signal. The microcontroller unit (MCU) controls the sensor signal via the I2C protocol of the sensing unit, acquires the data, and stores them in the flash memory. After the data collection is completed, the IEMS can be removed from the process chamber and the flash memory data can be analyzed via PC communication. We utilized the Origin application to visualize measured data using line graphs and contour maps. The IEMS block diagram is depicted in Figure 2.

**Figure 3:**
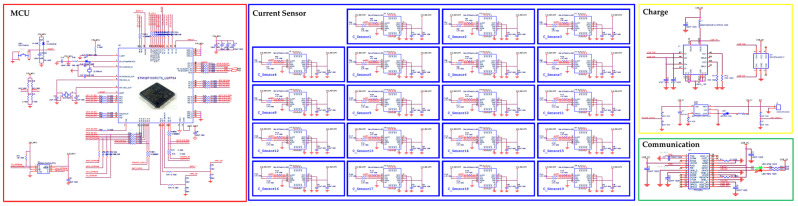
Circuit diagram of IEMS and peripheral circuit.

The IEMS was created utilizing the layout tools of Autodesk’s EAGLE PCB design software. The EAGLE logic tool was employed to design the IEMS circuit. Before drawing the circuit diagram, the actual environment and operational functions were considered when selecting the components. This includes an MCU for system-wide operation control, a charging circuit for charging the battery, and a data transmission and reception circuit. Figure 3 illustrates the circuit diagram of the IEMS.

IEMS employs EMI sheets to ensure stable operation within the plasma chamber. To protect circuits from electromagnetic waves, EMI sheets are inserted into each layer. To prevent metal oxidation and increase conductivity, the surface of the sensing component’s circuit is coated with gold.

Here, 19 sensing units are evenly distributed to ensure uniform measurement of the ion flux on the PCB’s surface. Six additional sensing units were placed around the central sensing unit on the PCB to provide information on the measurement uniformity of the current. In addition, as the size of the wafer increases, controlling the ion flux in the edge area has a substantial effect on the process yield; therefore, 12 sensing units are positioned to measure the ion flux in the edge area.

**Figure 4:**
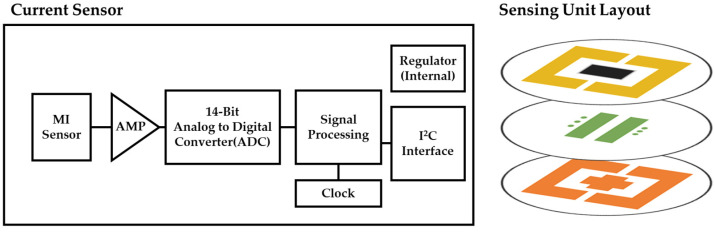
Block diagram of current sensor and PCB layout of sensing unit.

**Figure 5:**
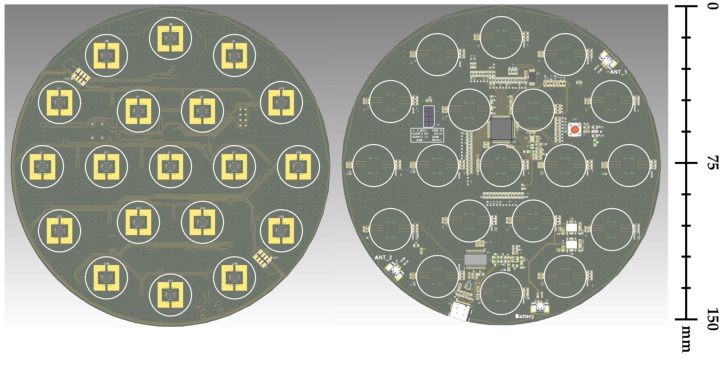
Hardware design of IEMS.

As the sensing component, coreless current sensors are typically shielded to prevent measurement errors caused by external magnetic fields. Because the two sensors built into the sensor can simultaneously detect the magnetic field and cancel out the external magnetic field, only the target magnetic field can be detected. The structure of the sensing unit for measuring the ion energy distribution is depicted in Figure 4, while the hardware structure of the IEMS is depicted in Figure 5.

## 3. Experiment Detail

### Experiment Setup

**Figure 6:**
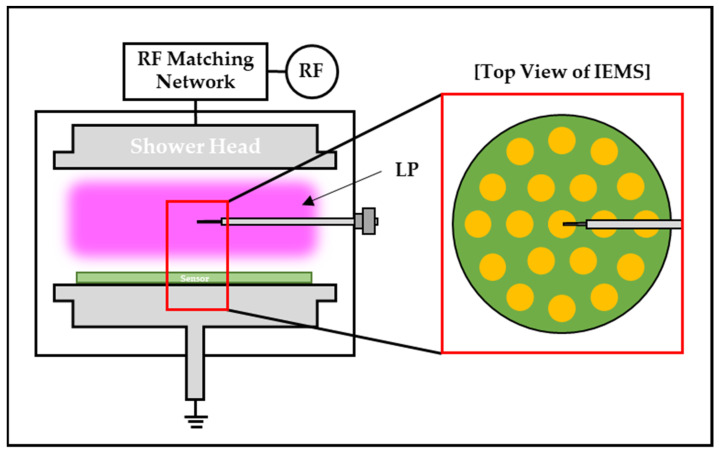
Ion current measurement using LP and IEMS.

Using the proposed wafer-type IEMS, ion currents were measured, and data were compared with LP for sensor reliability and validation. In the experiment, the results were compared based on radial position changes while adjusting the LP’s position, as depicted in Figure 6. Furthermore, we calibrated every sensor. Due to the structural limitations of the LP in use, radial position measurements were limited. Therefore, data were acquired at the center, 30 mm and 60 mm of the IEMS with the sensing unit. Table 1 shows the experimental conditions.

**Figure 7:**
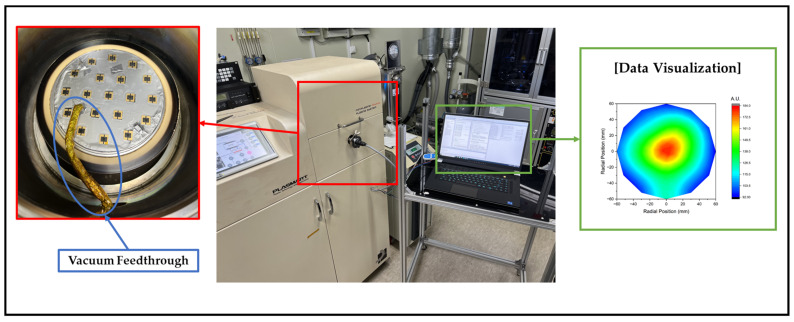
Schematic diagram of the experimental setup.

**Figure 8:**
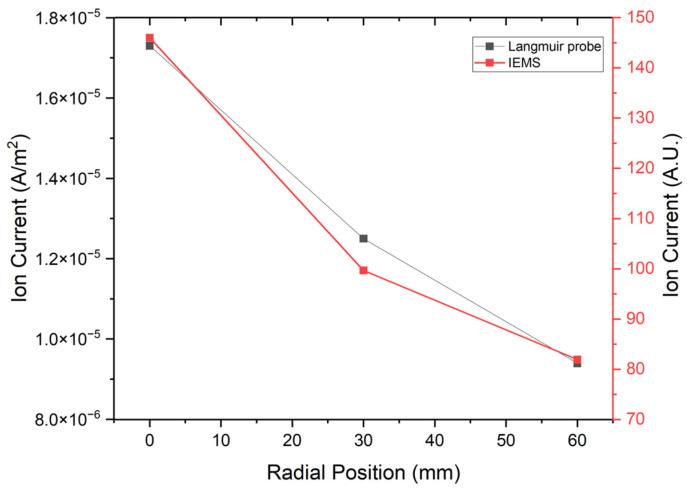
Comparison of the ion current using LP and IEMS. All data were acquired 30 s after plasma generation for stabilization.

As depicted in Figure 7, the experiment was conducted on a 6-inch ICP etcher. IEMS lithium polymer batteries generally expand in a vacuum. In this experiment, electricity was supplied externally through a vacuum feedthrough to ensure safety. Figure 8 illustrates the results of the experiment.

The results of ion current measurement according to the changes in radial position using IEMS and LP are similar. Moreover, as the radial position moved away from the center, the measured ion current tended to decrease. These results are identical to those of prior studies [29]. This demonstrates that the IEMS is operating normally in a plasma environment and that the experimental results for LP are accurate. Based on the results of this experiment, additional tests were conducted.

## 4. Results and Discussion

To confirm that IEMS can detect changes in ionic current caused by pressure and RF power, additional experiments were conducted. The pressure impact experiment was conducted by maintaining the RF power at 100 W and the Ar gas flow rate at 30 sccm, while increasing the Ar gas pressure from 30 mTorr to 70 mTorr in 20 mTorr increments. In addition, the experiment was conducted by increasing the RF power from 100 W to 400 W at 50 mTorr pressure and 30 sccm Ar flow rate. Table 2 and Table 3 illustrate the two experimental conditions.

**Figure 9:**
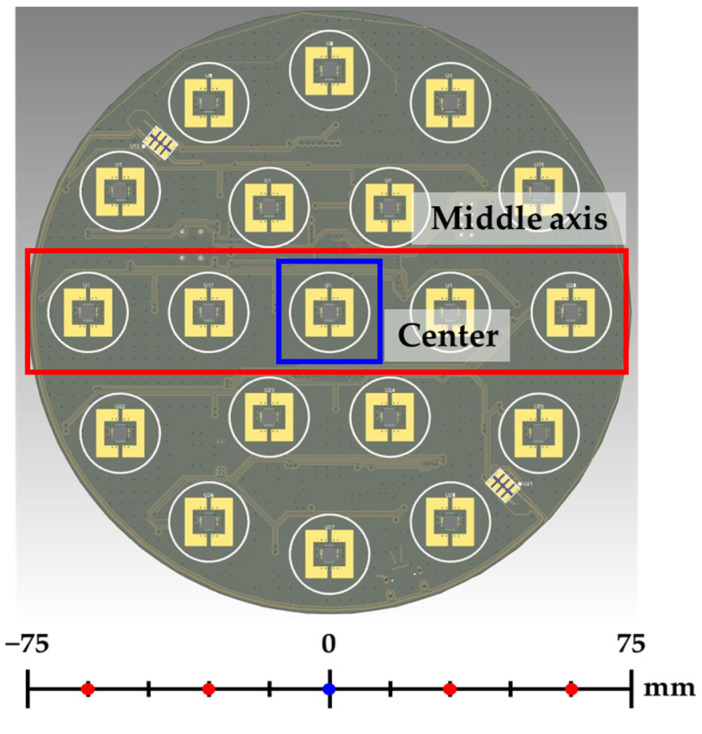
IEMS Sensing unit position.

As shown in Figure 9 and Table 4, the experimental results were visualized only for the results measured on the middle axis and center of the IEMS.

**Figure 10:**
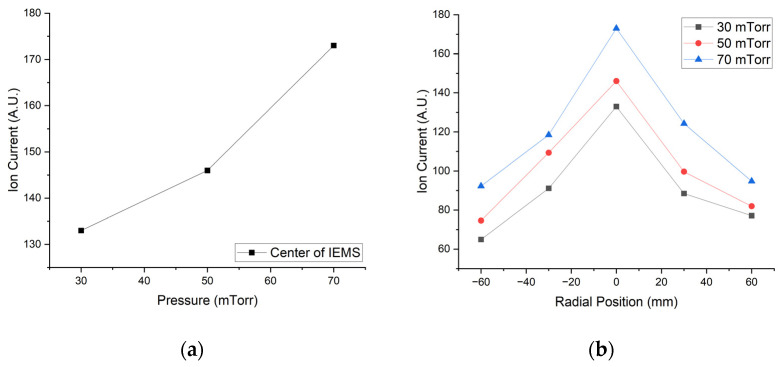
Experimental result according to pressure change; (**a**) Center position and (**b**) Middle axis. All data were acquired 30 s after plasma generation for stabilization.

**Figure 11:**
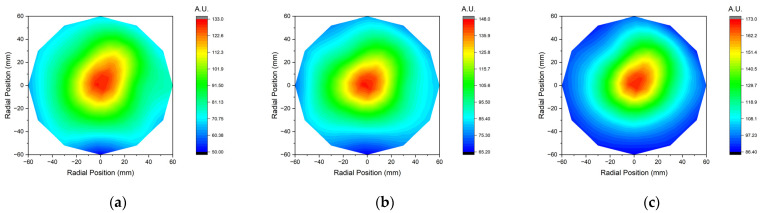
Contour map of ion current measured by IEMS according to pressure; (**a**) 30 mTorr, (**b**) 50 mTorr and (**c**) 70 mTorr. All data were acquired 30 s after plasma generation for stabilization.

Figure 10 and Figure 11 show the experimental results according to the pressure change using IEMS.

**Figure 12:**
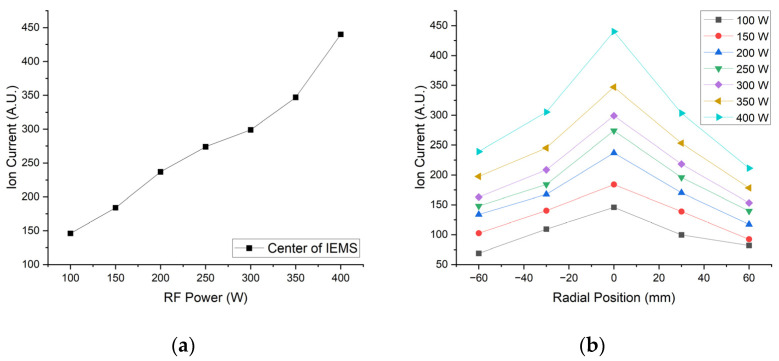
Experimental result according to varying RF power; (**a**) Center position and (**b**) Middle axis. All data were acquired 30 s after plasma generation for stabilization.

**Figure 13:**
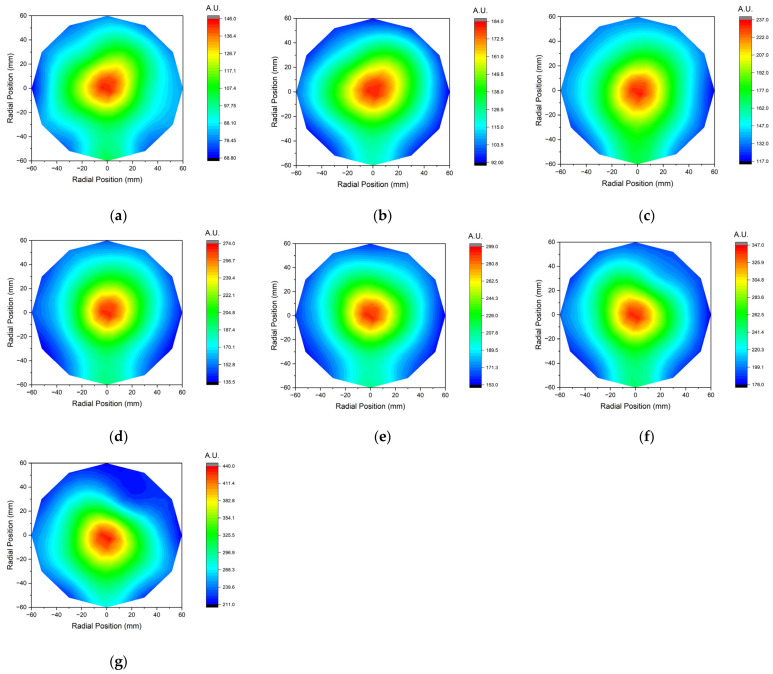
Contour map of ion current measured by IEMS according to RF power; (**a**) 100 W, (**b**) 150 W, (**c**) 200 W, (**d**) 250 W, (**e**) 300 W, (**f**) 350 W, and (**g**) 400 W. All data were acquired 30 s after plasma generation for stabilization.

Figure 12 and Figure 13 show the experimental results according to the RF power change using IEMS.

As a result of the experiment, the IEMS can detect the change in ion current according to the change in pressure and RF power. We explain the calculation of the ion energy in Section 2.1. Among them, through the sheath thickness calculation equation, as the pressure increases, the sheath thickness decreases, and the ion current increases, and the ion current increases as the RF power increases. Through this, it was possible to predict the trends of the ion current, and the result was the same. Furthermore, the IEMS operates normally in a plasma environment and is not damaged. This allowed us to confirm the validity and reliability of this sensor.

## 5. Conclusions

A wafer-type IEMS capable of measuring the spatial distribution of ion energy in a plasma chamber measuring 150 mm in diameter has been developed. IEMS can measure the distribution of ion current on a substrate, and the measurement result can be visually analyzed to determine the distribution of the ion current.

The IEMS was fabricated on a PCB. The IEMS does not necessitate any modifications to the chamber or chuck. Moreover, the system design does not necessitate semiconductor manufacturing processes. This results in relatively low manufacturing costs. In addition, it is simple to customize and expand the circuit.

In this experiment, the distribution of ion current in relation to varying Ar gas pressure and RF power was monitored and measured. To confirm the validity of the proposed sensor, the trends were compared to the LP. Validity was thus confirmed. In addition, IEMS functions normally in a plasma environment and detects ion current according to the process conditions. Through previous experiments, we confirmed the stability and effectiveness of this sensor. We will improve the completeness by supplementing some problems, and we will expand it to 300 mm wafer size so that it can be applied to various processes. If this sensor is applied in the actual manufacturing process, it will bring positive results to yield improvement and process prediction.

## Figures and Tables

**Table 1:** Experimental conditions for radial position change.

Parameter (Unit)	Value		
Radial position (mm)	0	30	60
Pressure (mTorr)	50		
RF power (W)	100		
Ar flow rate (sccm)	30		

**Table 2:** Experiment conditions for pressure change.

Parameter (Unit)	Value		
Pressure (mTorr)	30	50	70
RF power (W)	100		
Ar flow rate (sccm)	30		

**Table 3:** Experiment conditions for RF power change.

Parameter (Unit)	Value						
Pressure (mTorr)	100	150	200	250	300	350	400
RF power (W)	50						
Ar flow rate (sccm)	30						

**Table 4:** Radial position of IEMS sensing unit at middle axis and center position.

Parameter (Unit)	Value				
Radial position (mm)	−60	−30	0	30	60

## Data Availability

The datasets generated during the current study are available from the authors on reasonable request.

## References

[1] Gottscho R.A., Jurgensen C.W., Vitkavage D.J. (1992). Microscopic uniformity in plasma etching. J. Vac. Sci. Technol. B Microelectron. Nanometer Struct. Process. Meas. Phenomena.

[2] Adamovich I., Baalrud S.D., Bogaerts A., Bruggeman P.J., Cappelli M., Colombo V., Czarnetzki U., Ebert U., Eden J.G., Favia P. (2017). The 2017 Plasma Roadmap: Low temperature plasma science and technology. J. Phys. D Appl. Phys..

[3] Hayashi H., Kurihara K.K.K., Sekine M.S.M. (1996). Characterization of highly selective SiO2/Si3N4 etching of high-aspect-ratio holes. Jpn. J. Appl. Phys..

[4] Wang S.B., Wendt A.E. (2000). Control of ion energy distribution at substrates during plasma processing. J. Appl. Phys..

[5] Bruce R.H., Reinberg A.R. (1982). Profile Control with D-C Bias in Plasma Etching. J. Electrochem. Soc..

[6] Sobolewski M.A., Wang Y., Goyette A. (2002). Measurements and modeling of ion energy distributions in high-density, radio-frequency biased CF 4 discharges. J. Appl. Phys..

[7] Eriguchi K. (2017). Modeling of defect generation during plasma etching and its impact on electronic device performance—Plasma-induced damage. J. Phys. D Appl. Phys..

[8] Gahan D., Dolinaj B., Hopkins M.B. (2008). Retarding field analyzer for ion energy distribution measurements at a radio-frequency biased electrode. Rev. Sci. Instrum..

[9] Akatsuka H. (2019). Optical Emission Spectroscopic (OES) analysis for diagnostics of electron density and temperature in non-equilibrium argon plasma based on collisional-radiative model. Adv. Phys. X.

[10] Lee Y.I., Song W.S., Hong S.J. (2020). In situ monitoring of plasma ignition step in capacitively coupled plasma systems. Jpn. J. Appl. Phys..

[11] Evdokimov K.E., Konischev M.E., Pichugin V.F., Sun Z. (2017). Study of argon ions density and electron temperature and density in magnetron plasma by optical emission spectroscopy and collisional-radiative model. Resour.-Effic. Technol..

[12] Arshad M.Z., Jo K.J., Kim H.G., Hong S.J. (2018). Optical in situ monitoring of plasma-enhanced atomic layer deposition process. Jpn. J. Appl. Phys..

[13] Arshad M.Z., Hong S.J. (2018). In-situ detection method of abnormal plasma discharge in plasma-assisted deposition processes. Trans. Electr. Electron. Mater..

[14] An S.R., Choi J.E., Hong S.J. (2021). In-situ process monitoring for eco-friendly chemical vapor deposition chamber cleaning. J. Korean Phys. Soc..

[15] Matsutani A., Ohtsuki H., Koyama F., Iga K. (2000). Plasma diagnostics in inductively coupled plasma etching using Cl2/Xe. Jpn. J. Appl. Phys..

[16] Burlacov I., Börner K., Spies H.J., Biermann H., Lopatik D., Zimmermann H., Röpcke J. (2012). In-situ monitoring of plasma enhanced nitriding processes using infrared absorption and mass spectroscopy. Surf. Coat. Technol..

[17] Lee N., Kwon O., Chung C.W. (2021). Correlation of RF impedance with Ar plasma parameters in semiconductor etch equipment using inductively coupled plasma. AIP Adv..

[18] Kang G.G., An S.R., Kim K.P., Hong S. (2019). An in-situ monitoring method for PECVD process equipment condition. Plasma Sci. Technol..

[19] Yokoshima I. (1993). RF impedance measurements by voltage-current detection. IEEE Trans. Instrum. Meas..

[20] Hopwood J., Guarnieri C.R., Whitehair S.J., Cuomo J.J. (1993). Langmuir probe measurements of a radio frequency induction plasma. J. Vac. Sci. Technol. A Vac. Surf. Film..

[21] Cox T.I., Deshmukh V.G.I., Hope D.A.O., Hydes A.J., Braithwaite N.S.J., Benjamin N.M.P. (1987). The use of Langmuir probes and optical emission spectroscopy to measure electron energy distribution functions in RF-generated argon plasmas. J. Phys. D Appl. Phys..

[22] Merlino R.L. (2007). Understanding Langmuir probe current-voltage characteristics. Am. J. Phys..

[23] Kim Y.D., Lee H.C., Chung C.W. (2013). A study on the maximum power transfer condition in an inductively coupled plasma using transformer circuit model. Phys. Plasmas.

[24] Irimiciuc S.A., Chertopalov S., Lancok J., Craciun V. (2021). Langmuir Probe technique for plasma characterization during pulsed laser Deposition process. Coatings.

[25] Kim J.H., Koo Y.S., Song W.S., Hong S.J. (2022). On-Wafer Temperature Monitoring Sensor for Condition Monitoring of Repaired Electrostatic Chuck. Electronics.

[26] Kawamura E., Vahedi V., Lieberman M.A., Birdsall C.K. (1999). Ion energy distributions in rf sheaths; review, analysis and simulation. Plasma Sources Sci. Technol..

[27] Langmuir I. (1913). The effect of space charge and residual gases on thermionic currents in high vacuum. Phys. Rev..

[28] Gahan D., Daniels S., Hayden C., Scullin P., O’sullivan D., Pei Y.T., Hopkins M.B. (2012). Ion energy distribution measurements in rf and pulsed dc plasma discharges. Plasma Sources Sci. Technol..

[29] Kim J.H., Kim Y.C., Chung C.W. (2015). Experimental investigation on plasma parameter profiles on a wafer level with reactor gap lengths in an inductively coupled plasma. Phys. Plasmas.

